# Efficacy of Zonisamide in Migraineurs with Nonresponse to Topiramate

**DOI:** 10.1155/2014/891348

**Published:** 2014-07-02

**Authors:** Jin-Young Chung, Min-Wook Kim, Manho Kim

**Affiliations:** ^1^Department of Veterinary Internal Medicine and Geriatrics, Kangwon National University, Gangwon-do, Republic of Korea; ^2^Institute of Catholic Integrative Medicine (ICIM), Incheon St. Mary's Hospital, Catholic University of Korea, Incheon, Republic of Korea; ^3^Department of Neurology, Seoul National University Hospital, 101 Daehakro, Chongno-ku, Seoul 110-744, Republic of Korea

## Abstract

This study investigated another type of carbonic anhydrase inhibitor and antiepileptic drug, zonisamide, in order to evaluate its potential effectiveness for migraine prophylaxis refractory to topiramate, and to assess intolerability to adverse events, paresthesia in particular. This is an open-labeled retrospective single center study. We included headache patients who met the requirement of migraine without aura and were refractory to topiramate. Patients were treated only with zonisamide 100 mg/day, directly switching from topiramate. Patients were monitored every month for three months. A positive response to treatment (responders) was defined as a 50% or greater reduction in headache days at three months after study commencement, compared with baseline. One hundred and twenty migraineurs who were refractory to topiramate were recruited. Compared with baseline, headache days with zonisamide showed a decrease, compared with baseline (*P* < 0.01). Two patients complained of adverse effects, such as paresthesia. These results suggest that zonisamide is effective for migraine prophylaxis refractory to topiramate, or intolerable patients due to topiramate-induced paresthesia.

## 1. Introduction

Migraine is a common neurologic disorder with a prevalence of 10 to 12% in western countries [1] and 8.4 to 22.3% in eastern countries [2]. Only a few drugs, including *β*-blockers, calcium-channel blockers, neuroleptics, antidepressants, and antiepileptics, have demonstrated effectiveness in migraine prophylaxis; however, their use is sometimes limited due to adverse effect [3].

Topiramate has been approved as an indication for some types of epileptic seizures. Its efficacy has also been showed in treatment of neuropathic pain, essential tremor, and some psychiatric conditions [4–6]. Topiramate has recently been approved as an evidence-based first-line drug for migraine prophylaxis in adults [7, 8].

Topiramate is a sulfamate-substituted monosaccharide and the expected mechanisms of topiramate are as follows. Four properties that may contribute to topiramate's antiepileptic and antimigraine efficacy include blockage of voltage-dependent sodium channels, augmentation of gamma-aminobutyrate acid activity at subtypes of GABA-A receptors, antagonism of the AMPA/kainate subtype of the glutamate receptor, and inhibition of the carbonic anhydrase enzyme, particularly isozymes II and IV [9].

However, migraineurs treated with topiramate frequently experience adverse reactions, including paresthesia, fatigue, memory difficulties, loss of appetite, and weight loss [10, 11]. As a result of these adverse events, a large number of migraineurs dropped out, mainly due to paresthesia, which is induced by inhibition of carbonic anhydrase [12].

Zonisamide, similar to topiramate, is a sulfonamide anticonvulsant and has been used in Japan for a long time for treatment of epilepsy. Zonisamide has recently been approved in other countries as an adjunctive therapy for partial seizures in adults [13]. Although the exact mechanism of action is unknown, it appears to be very similar to that of topiramate: blockade of voltage sodium channels, inhibition of carbonic anhydrase enzyme, enhancement of the release of GABA, modulation of serotoninergic and dopaminergic neurotransmission, and inhibition of potassium-mediated release of glutamate [14, 15]. Zonisamide, but not topiramate, also appears to block T-type calcium channels [16].

The purpose of this study was to overcome the migraine prophylaxis with zonisamide in patients refractory to topiramate because the limitations of topiramate in migraineurs were adverse reactions, such as paresthesia, fatigue, memory difficulties, loss of appetite, and weight loss [11, 12]. In this study, we determine potential effectiveness of zonisamide for migraine prophylaxis in patients refractory to topiramate and assess adverse events of zonisamide.

## 2. Materials and Methods

### 2.1. Study Objectives

The primary objective was to evaluate the efficacy of zonisamide for prophylaxis of migraine attacks in patients refractory to topiramate. The primary efficacy outcome was headache frequencies over 12 weeks. The secondary efficacy outcome was the incidence of adverse events. Therefore, the secondary objective was to determine the rate of adverse events in comparison with that of topiramate.

### 2.2. Including and Excluding Criteria

Consecutive patients with “migraine without aura,” who had been followed up in the headache clinic at Seoul National University, were enrolled. Inclusion criteria were as follows: (1) diagnosis of “migraine without aura” according to the criteria of the* International Classification of Headache disorders*, 2nd edition [17] at the time of topiramate treatment. Therefore, the migraineurs with less than 15 per month, that is, episodic migraine rather than chronic migraine, could be included when the zonisamide trial, (2) age 20 to 70 years, (3) the ability to read and understand the self-report scales, including the headache diary, used in this study, and (4) refractory to topiramate treatment defined as less than 50% reduction of headache for a three-month period of treatment with a tolerable dose (75 mg daily dose in Korea) [12].

Subjects with a history or the following conditions were excluded: (1) treatment with other antiepileptic drugs, (2) treatment with migraine prophylactic medications other than topiramate, (3) less than a three-month duration of a topiramate trial, (4) history of allergic response to topiramate, and (5) past history of impaired hepatic or renal function; an abnormal electrocardiography; a psychiatric disorder; a history of substance abuse; pregnancy or lactation; use of antipsychotics, antidepressants, or antianxiety drugs during the 4-week period prior to commencement. However, patients with topiramate who had previously experienced a topiramate-induced adverse event but maintained this drug were not excluded. Patients who did not take the medication on less than 80% of scheduled days were excluded from the study.

### 2.3. Study Design and Protocol (Figure 1)

This study was designed as nonrandomized, retrospective, and open-label study. Patients were treated with only zonisamide 100 mg/day, directly switching from topiramate. Zonisamide was administered in two divided doses. Patients were educated to allow taking acute rescue medication for the migraine attack and were asked to record this in the headache diary. All patients were asked to maintain a headache diary, in which details on the number of headaches and adverse events were recorded. Baseline headache frequencies were obtained from headache diaries for the baseline phase. Patients were monitored every month up to three months. A positive response to treatment (responders) was defined as a 50% or greater reduction in headache frequency at three months after study commencement, compared with baseline, from which therapeutic efficacy of zonisamide on migraine prevention was analyzed. The study was approved by the Institutional Review Board of Seoul National University Hospital.

### 2.4. Statistical Analysis

Intragroup comparison for headache frequencies, Wilcoxon Rank Sum test, was performed. Frequencies of side effects were compared using Fisher's exact test. *P* values were two-tailed and statistical significance was accepted for *P* values less than 0.05.

## 3. Results

### 3.1. Description of Participants

A total of 120 migraineurs (92 women and 28 men) with refractory to topiramate were initially included in the study. The average age for this group was 52.0 ± 11.0 years, ranging from 20.0 to 80.0 years (Table 1). At first month recruitment, a total of 85 patients completed a one-month diary and participated in this study.

Fifty-five patients completed three-month treatment. Eighty-five patients (M : F = 17 : 68) were evaluated after one month with zonisamide treatment.

### 3.2. Adverse Events

Retrospective evaluation of reasons for dropout was confirmed by medical records or a telephone call to the patient. Reasons that patients did not make follow-up visits included the following: no effect or aggravation of headache (17%), side effects (12%; dizziness, light headedness, nausea, insomnia, and drowsiness), rapid improvements that caused the patient not to want to visit the clinic (23%), visit to another physician (5%), or undetermined (42%). None of the patients complained of paresthesia, except for undetermined patients whom the researcher was unable to contact, or patients could not recall the exact reasons.

At the first month, 35 patients were dropped with the following reasons: no effect or aggravation of headache (*n* = 7), side effect (*n* = 7; dizziness 3, light headedness 1, nausea 1, insomnia 1, and drowsiness 1), rapid improvement that caused the patient not to want to visit the clinic (*n* = 6), visit to another physician (*n* = 2), or unknown (*n* = 13). At the second month, 35 patients were dropped with the following reasons: no effect or aggravation of headache (*n* = 5), side effect (*n* = 1; nausea 1), rapid improvement that caused the patient not to want to visit the clinic (*n* = 10), visit to another physician (*n* = 2), or unknown (*n* = 17). At the third month, 5 patients, who were dropped with unknown reasons at the second month, were included again in the recruits of migraineur.

At three months, 55 patients (10 men, 45 women) were followed up. Subjects rarely complained about medication related adverse events, such as paresthesia, memory disturbance, loss of appetite, or abdominal discomfort, which are known as the main adverse events associated with topiramate. In this study, only two of them complained of side effect (nausea 1, dizziness 1) who completed this trial. In the topiramate group reported previously in the same clinic [12]. The profiles of adverse events using topiramate were paresthesia (54.9%, 73/133), fatigue (44.4%, 59/133), cognitive dysfunction (32.3%, 43/133), diminished appetite (18.0%, 24/133), taste perversion (11.3%, 15/133), and nausea (2.3%, 3/133).

### 3.3. Headache Frequencies and Responder Rate Evaluation

**Figure 2:**
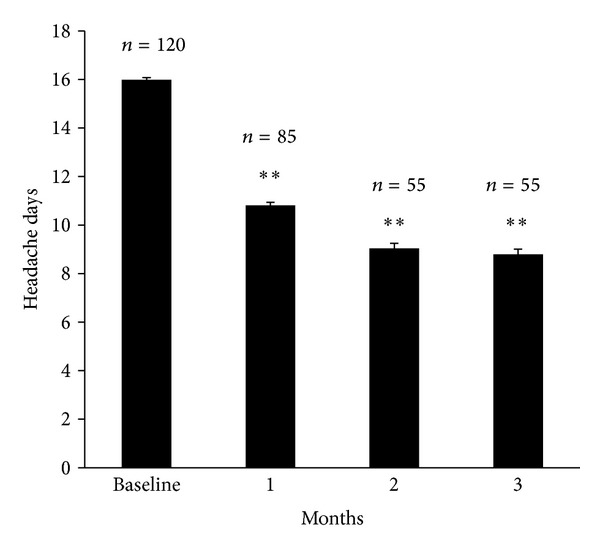
Headache days with zonisamide treatment showed a decrease at every month, compared with baseline (*P* < 0.01).

During baseline evaluation of subjects refractory to topiramate, the mean headache frequency was 16.0 ± 10.8 per month. At the first month, monthly headache frequencies decreased to 10.8 ± 10.8 in total. At the second month, headache frequency was 9.0 ± 10.7. The third month evaluation showed 8.8 ± 11.2. Headache frequencies with zonisamide treatment showed a decrease at every month, compared with baseline (*P* < 0.01, Wilcoxon Rank Sum test). The most effective reduction of migraine frequencies was shown for the first month (Figure 2).

## 4. Discussion

In this study, we investigated the potential efficacy of zonisamide for migraineurs who had not enough response with topiramate. In addition, we also determined development of paresthesia by zonisamide, which is the most common adverse event that inhibits migraineurs from maintaining topiramate. Results showed that zonisamide shows further effectiveness in patients who are refractory to topiramate. In addition, none of the patients developed paresthesia, the most troubling adverse event associated with topiramate. Our study supports zonisamide as a potentially effective medication for migraine prophylaxis.

Topiramate is the first-line drug for prevention of migraine and is also effective for patients who are refractory to other medications or those with chronic migraine. Topiramate is known in the Western setting to be effective in migraine prevention with dosages at or above 100 mg/day. However these previous studies could not cover the Asian population. Some studies showed that lower dosage of topiramate is effective in migraine prevention with lower adverse reactions, particularly in the Asian population [12, 18–20]. Unfortunately, quite a large number of patients cannot tolerate topiramate. The limitations of topiramate in migraineurs were adverse reactions, such as paresthesia, fatigue, memory difficulties, loss of appetite, and weight loss [11, 12], and paresthesia was the most common cause of dropout. This paresthesia may result from inhibition of carbonic anhydrase, lowering the potassium level in serum. Mechanisms of zonisamide are similar to those of topiramate; therefore, similar adverse reactions are expected [14–16]. One report showed that fifteen patients (24%) had adverse effects, including concentration difficulties, fatigue, and paresthesia, by zonisamide in migraineurs refractory to topiramate [21]. Another report showed that only 4 patients (12%) reported adverse effects, including difficulty concentrating and mood disorders [22]. However, these symptoms were transient and tolerable within the titration period [21, 22].

Of particular interest, in this study, adverse events were low. One possibility to account for this less side effect might be due to selection. The patients using topiramate with uncomfortable side effect had been dropped out. Thus, subjects may not be susceptible to this adverse event. In addition, paresthesia is usually transient; thus, patients who used topiramate for longer than one month usually did not suffer from adverse events. In this study, we changed zonisamide in patients who were refractory to topiramate, at least maintaining topiramate for three months. Transient adverse events induced by carbonic anhydrase inhibition might not occur during medication with zonisamide, at least at this dose level. The other possibility could be that zonisamide, which blocks T-type calcium channels [16], which is different from topiramate, may have a potential role in migraine prophylaxis [21].

This study was an open-label study; the results are of limited value, especially given the universally high placebo response in all headache trials. It should be validated by a proper double-blind randomized controlled trial against placebo. In our study, direct switching from topiramate to zonisamide was done because the patients had already been exposed to a similar type of medication. Neither a gradual dosing up protocol nor overlapping with topiramate was attempted. The outcome of switching protocol did not result in any serious or troublesome unwanted events. However, this study could not provide information on whether a dose-up schedule or overlapping protocol. It suggests a possibility that zonisamide can be used as the first drug or changed from a prophylactic medication other than topiramate. In addition, the treatment dose of zonisamide was unchanged in our study, 100 mg/day divided by two times. It is unknown whether this dose is optimal or not for the prophylaxis of migraine. The dose can be different by ethnic group. For example, an increased dose of topiramate, up to 100 mg/day, is usually recommended. However, in Korea, only a few patients can tolerate 100 mg/day, and most patients gave up during the dosing up schedule, especially 50 to 75 mg [12]. Therefore, whether a zonisamide dose of 100 mg/day is optimal or not has not been determined. At least, in our study, zonisamide showed further benefits, reducing headache frequency with more than 50% responder rate and with less adverse events. Number of pain killers per month must be an indicator for the efficacy of zonisamide. The patients may take more analgesics to manage the attack or may not take the medication when the attack is mild degree. Therefore, in addition to the headache frequency, number of taking the acute medication can reflect the effectiveness of zonisamide on the management of headache as well as the severity of migraine attack. However, lack of data about the number of acute treatments per month is the major limitation of this study.

In most cases, three-month evaluation is the most common protocol for determination of the efficacy of candidate medication. In our case, we found that even during the first month, a rapid reduction of headache frequency was reported by more than 50% of responders. This rapid response can be one of the reasons that patients did not make a follow-up visit. In our study, 23% of patients who did not make a follow-up replied that they did not need further treatment because of improvement of their condition. After a rapid response for the first one-month period, the reduction rate appeared to have slowed or remained thereafter.

Zonisamide is similar to topiramate but has not yet been approved as an antimigraine medicine. In conclusion, results of this study suggest that zonisamide may be an effective agent for migraine prophylaxis. They could be compatible with previous studies of zonisamide treatment in migraine patients [21, 22]. It can be suggested that zonisamide is one of the candidates for a rapid reduction in headaches, particularly for cases of topiramate-refractory migraine.

## Figures and Tables

**Figure 1:**
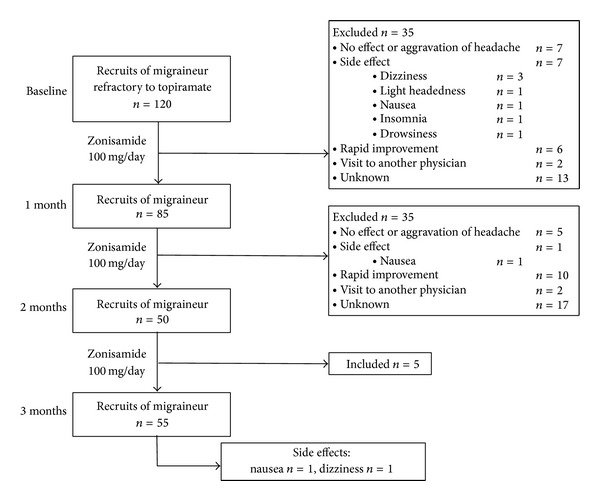
Study design and protocol. Patients were treated with only zonisamide 100 mg/day, directly switching from topiramate.

**Table 1:** Disposition of patients.

	Total patients
Patients (*n*)	120
Mean age	52.0 ± 11.0
Sex (M/F)	28/92
Baseline headache days	15.99 ± 10.85
